# Effect of scar distribution on transmural and planar repolarization gradients and dispersion in non-ischemic cardiomyopathies with ventricular arrhythmias

**DOI:** 10.1093/europace/euaf244

**Published:** 2025-12-02

**Authors:** Johanna B Tonko, Eva Cabrera-Borrego, Pablo Sánchez-Millán, Juan Jiménez-Jáimez, Anthony Chow, Pier D Lambiase

**Affiliations:** Institute for Cardiovascular Science, University College London, 5 University Street, London WC1E 6JF, UK; Department for Cardiology, St Bartholomew’s Hospital, W Smithfield, London EC1A 7BE, UK; Department for Cardiology, Virgen de Las Nieves Hospital, Av. de las Fuerzas Armadas, Granada 18014, Spain; Department for Cardiology, Virgen de Las Nieves Hospital, Av. de las Fuerzas Armadas, Granada 18014, Spain; Department for Cardiology, Virgen de Las Nieves Hospital, Av. de las Fuerzas Armadas, Granada 18014, Spain; Institute for Cardiovascular Science, University College London, 5 University Street, London WC1E 6JF, UK; Department for Cardiology, St Bartholomew’s Hospital, W Smithfield, London EC1A 7BE, UK; Institute for Cardiovascular Science, University College London, 5 University Street, London WC1E 6JF, UK; Department for Cardiology, St Bartholomew’s Hospital, W Smithfield, London EC1A 7BE, UK

**Keywords:** Repolarization Dispersion, Repolarization Gradients, Non-ischemic Cardiomyopathy, Cardiac MRI, Myocardial Scar, Ventricular Arrhythmias

Ventricular tachycardias (VT) in non-ischemic cardiomyopathies (NICM) arise from a complex interplay between structural myocardial changes and dynamic electrophysiological properties. Yet, conventional substrate mapping strategies and imaging^1,2^ remain focused on identifying fixed structural and/or conduction abnormalities, often neglecting other functional elements such as repolarization changes.^3^

Altered repolarization contributes to increased susceptibility to re-entrant VTs in ischemic heart disease,^4,5^ yet its proarrhythmogenic role in NICM is poorly characterized. Moreover, the impact of transmural scar distribution on 3-dimensional repolarization patterns remains underexplored due to the restriction of conventional mapping approaches to endo- and epicardial surfaces.

This study aimed to assess transmural and planar repolarization heterogeneity in NICM, stratified by myocardial scar pattern, by integrating electro-anatomical mapping (EAM) with delayed enhancement MRI (DE-MRI) in patients undergoing clinical VT ablation.

## Methods

Twenty-seven patients who underwent endo-epicardial VT ablation (EnSite™ X, Abbott) with pre-procedural MRI demonstrating non-ischemic scar patterns were retrospectively reviewed. Cases with isolated right ventricular scar, dual pathology, or inadequate mapping density were excluded.

**Figure 1:**
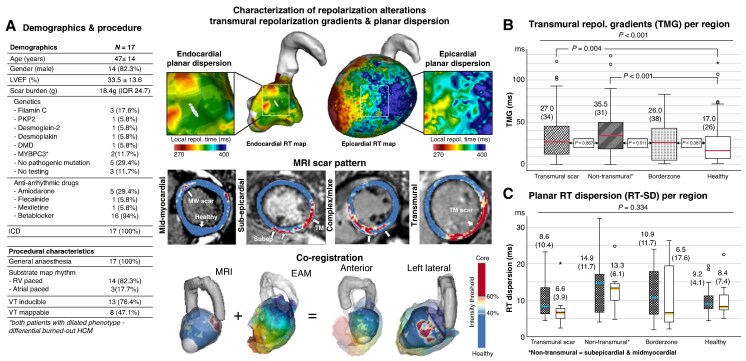
Repolarization alterations in non-ischemic substrates. Left: baseline demographics and procedural characteristics. Middle-Top: (*A*) Example of 3D endo-epicardial repolarization map with zoom-in at sites of local dispersion (epicardium > endocardium). Middle-Mid: Examples of MRI scar pattern re-constructed with Adas3D^TM^. Middle-Bottom: Example of co-registration of MRI and electro-anatomical maps based on anatomical landmarks. Right: Top (*B*) Box plots for transmural RT gradients with median [IQR] per region and results of Kruskal Wallis test (non-transmural region includes both sub-epicardial and mid-myocardial scar regions). Bottom (*C*) Box plots for planar dispersion shows average RT-dispersion per region (median [IQR]) plotted separately for endo- and epicardium, respectively.

Repolarization maps were generated as previously reported^6^ employing the Wyatt method.^7,8^ DE-MRI images were segmented in Adas3D™ to define regions of transmural, subepicardial, mid-myocardial scar, border zone, and remote myocardium. Repolarization maps were co-registered with MRI using anatomical landmarks (*Figure 1*).

Opposing endo-epicardial EGM pairs were manually identified and transmural repolarization gradients (RT-TMG) calculated as the difference between epicardial and endocardial RTs for each pair. Planar repolarization dispersion was estimated as the standard deviation of RTs (RT-SD) within 10 mm to each other, separate for each surface and MRI region. Kruskal-Wallis tests were used to compare RT-metrics across MRI-defined regions; endocardial vs. epicardial RTs were compared using Wilcoxon signed-rank test. Statistical significance was set at *α* = 0.05 and corrected for multiple tests (Bonferroni) (IBM SPSS 29).

## Results

Seventeen patients (age 47.5 ± 14years; LVEF 33.5 ± 13.6%) were included. Baseline and procedural characteristics are reported in *Figure 1A*.

RT-TMGs were estimated for 525 endo-epicardial EGM pairs. The median RT-TMG including all EGM pairs in all segments was 26.0 ms (IQR 33.0, range 0–163 ms). Comparison across MRI-defined segments revealed significant differences between regions (*P* < 0.001). On average, the steepest gradients were observed in subepicardial scar (36.5 ms [IQR 36.0]), significantly higher than in remote normal myocardium (17.0 ms [IQR 26.0], *P* < 0.001) and border-zone (26.0 ms [IQR 38.0], *P* = 0.011) and non-significantly higher than in transmural scar (27.0 ms [IQR 34.0], *P* = 0.867) (*Figure 1B*).

Average epicardial RT was significantly shorter than endocardial RT in normal myocardium (epi 312.0 ms [IQR 93.0] vs. endo 332.0 ms [IQR 72.0], *P* < 0.001). Yet, over areas of subepicardial (epi 372.5 ms [IQR 96.0] vs. endo 354.0 ms [IQR 64.0], *P* < 0.001) and transmural scar (epi 392.0 ms [IQR 86.0] vs. endo 376.0 ms [IQR 62.0], *P* < 0.001) the pattern was reversed.

Highest dispersion was recorded over areas of non-transmural scar on the epicardium (14.9 ms [IQR 11.7]), lowest over remote areas [9.2 ms (4.1)]. Generally, epicardial dispersion exceeded endocardial dispersion in transmural, non-transmural, and border zone segments, but was similar in remote normal myocardium. However, overall there was no significant difference in planar dispersion across regions (*P* = 0.334) (see *Figure 1C* for segmental results). Substantial inter- and intra-individual variability was observed in both transmural and planar RT-metrics.

In eight patients, a strategic VT map had been acquired; in five of these cases diastolic/presystolic potentials localized to sites of scar or border zone of the LV free wall, permitting association with endo-epicardial RT metrics. At these sites, average RT-TMGs were 34.0 ms [IQR 29] with maximum values up to 90 ms; planar dispersion was 22.6 ms [IQR 25.5]. However, the absence of combined endo-epicardial VT mapping data precluded a direct correlation of TMGs with 3D re-entry circuit paths.

## Discussion

This study provides novel in-vivo evidence of transmural and planar repolarization heterogeneity in patients with NICM dependent on myocardial scar pattern. Subepicardial and transmural scar regions were associated with significantly elevated RT-TMGs and reversal of epi-to-endocardial repolarization gradients compared with normal myocardium. These changes likely reflect combined effects of prolonged local action potential duration and disruption of electrotonic coupling across myocardial layers due to fibrotic tissue.

The observed increase in epicardial dispersion compared with endocardium, including in transmural scar segments, suggests a more advanced electrical remodelling of subepicardial layers in non-ischemic substrates also at sites with transmural myocardial disease. RT-dispersion was highest at sites associated with VT.

A notable finding of this study was a wide variation in repolarization metrics both on an intra-segmental as well as inter-individual level. The average gradient and dispersion values were modest, but individual segments often displayed high *intra*-segmental heterogeneity harbouring localized sites of steep gradients, that may facilitate re-entry. In turn, the high *inter*-individual variability among patients may reflect the general geno- and phenotypic heterogeneity of this NICM cohort supporting the need for personalized electrophysiological profiling in addition to integration of genetic and imaging data.

Lastly, this study also reinforces the limitations of current clinical substrate mapping strategies, which often lack the resolution or dimensionality to capture transmural activation- and repolarization dynamics. The significantly elevated transmural repolarization gradients notably in sub-epicardial scar and at sites of VT underscore the need for incorporating 3D functional information when assessing re-entry vulnerable sites. Indeed, the sites with highest susceptibility for organized re-entrant arrhythmias or wave break may only be unmasked with transmural measurements.

### Limitations

The sample size was small in a genetically diverse cohort, restricted to left-ventricular phenotypes, and without standardized pacing site, limiting generalizability. In particular, number of cases with mappable VT to relate to repolarisation alterations were sparse.

Unipolar EGMs are susceptible to far-field contamination and distortion requiring exclusion, reducing point density, complicating TMG estimation and limiting the sensitivity of dispersion metrics. Co-registration of EAM/MRI models may introduce spatial inaccuracies, and DE-MRI underestimate diffuse interstitial abnormalities.

All procedures were performed under general anaesthesia, which reduces sympathetic tone and may blunt dynamic repolarization changes. Furthermore, beat-to-beat and cycle length-dependent variability were not assessed due to methodological constraints; this should be addressed in future prospective work.

## Conclusion

In NICM, subepicardial and transmural scar were associated with elevated transmural repolarization gradients and increased epicardial dispersion. These findings support the hypothesis that structural fibrosis may influence repolarization dynamics in a regional and layer-specific manner. Given the observed inter-individual variability, comprehensive 3D repolarization mapping warrants further investigation as a potential tool for personalized arrhythmogenic substrate characterization and identification of re-entry vulnerable sites in NICM.

## Data Availability

Due to privacy restriction, data cannot be shared directly with third parties.
